# Four-Week Hypofractionated Radiotherapy for Early Glottic Cancer: A Prospective Study of Toxicity and Tolerance With Historical Comparison

**DOI:** 10.7759/cureus.97482

**Published:** 2025-11-22

**Authors:** Georgios Moschos, Alexia Theodoridou, Areti Gkantaifi, Georgios Giakoumettis, Antonio Capizzello, Styliani Stylianidou, Georgios Plataniotis

**Affiliations:** 1 Department of Radiation Oncology, American Hellenic Educational Progressive Association (AHEPA) University Hospital, Thessaloniki, GRC

**Keywords:** early glottic cancer, hypofractionation, radiation oncology, toxicity, volumetric‐modulated arc therapy (vmat)

## Abstract

Introduction: Hypofractionated radiotherapy has become an increasingly accepted treatment for early-stage glottic carcinoma, aiming to optimize tumor control while minimizing the impact of accelerated repopulation and treatment prolongation. However, evidence regarding more hypofractionated regimens with fraction sizes above 2.5 Gy remains limited, particularly for T2 disease.

Methods: We conducted a prospective interventional study at the Department of Radiotherapy, American Hellenic Educational Progressive Association (AHEPA) University Hospital, including 23 patients, so far, with histologically confirmed T1-T2N0M0 glottic carcinoma treated with definitive hypofractionated radiotherapy. Treatment consisted of 55 Gy in 20 fractions (2.75 Gy/fraction) delivered with volumetric modulated arc therapy (VMAT) and daily image guidance. The primary endpoint was toxicity; local control was a secondary endpoint, though its evaluation is limited by the relatively short follow-up. Results were also descriptively compared with prior institutional experience using conventional or mildly hypofractionated three-dimensional conformal radiotherapy.

Results: At a median follow-up of 16 (range: 1-36) months, 22 of 23 (95.7%) patients achieved a complete response, while one patient (4.3%) had residual disease and underwent successful salvage transoral laser surgery. Two patients experienced local recurrence after initial complete response; both were successfully salvaged surgically and remain disease-free. By Kaplan-Meier analysis, the one-year local control was 91.7% for T1 disease (95% CI: 76.0-100.0%) and 85.7% for T2 disease (95% CI: 59.8-100.0%), corresponding to an overall one-year local control of 91.3% (log-rank p=0.65). All patients were alive at the last follow-up. Acute toxicities were primarily grade 1-2 (larynx, mucosa, skin, esophagus). Grade 3 acute events included hoarseness (n=7) and dysphagia requiring temporary enteral feeding (n=3). Late toxicities were generally mild, with most events grade 1; grade 2 toxicities involved skin, esophagus, or larynx in a few patients. No grade 4 or 5 events occurred.

Conclusions: Hypofractionated VMAT with 55 Gy in 20 fractions is a feasible and safe approach for early glottic carcinoma, providing very good tumor control in T1 disease, acceptable outcomes in T2 disease, and a low incidence of significant toxicity. These findings are consistent with international evidence and compare favorably with prior institutional audit results. Longer follow-up and larger cohorts are required to confirm the durability of local control, particularly in T2 disease.

## Introduction

Head and neck squamous cell carcinoma represents the seventh most common cancer worldwide [1]. Laryngeal carcinoma accounts for approximately 1-2% of all cancers, with early-stage glottic disease being the most frequent presentation [2].

Radiotherapy remains a standard curative modality, aiming at excellent tumor control while preserving voice and swallowing function [3]. Conventionally fractionated radiotherapy (2 Gy per fraction, 60-70 Gy over six to seven weeks) has been the standard of care. However, prolonged treatment increases the risk of accelerated tumor repopulation, with an estimated daily loss of tumor control requiring additional compensatory dose [4-6]. Hypofractionated regimens (2.2-3.0 Gy per fraction, three to five weeks) are particularly attractive as they shorten overall treatment time, minimize repopulation, thereby enhancing biological effectiveness, while it is much more convenient for patients, especially those traveling from remote areas for their treatment [7].

The introduction of advanced radiotherapy techniques, such as volumetric modulated arc therapy (VMAT), allows highly conformal dose delivery with improved sparing of normal tissues, substantially reducing acute and late toxicity compared with older techniques. This makes hypofractionation more feasible and potentially safer in the management of early-stage glottic cancer [8].

The evidence base for hypofractionation is strongest in T1 glottic cancers, whereas data in T2 disease are sparse. According to the Royal College of Radiologists guidelines, the published experience with hypofractionated regimens is still insufficient, underlining the importance of further prospective studies [9].

The aim of this prospective phase I-II study was to evaluate the safety, tolerability, and early efficacy of a four-week hypofractionated VMAT regimen (55 Gy in 20 fractions) for patients with T1-T2N0M0 glottic carcinoma. The primary endpoint was treatment-related toxicity, and the secondary endpoint was local control, compared with the institution’s prior experience using conventional or mildly hypofractionated three-dimensional conformal radiotherapy (3D-CRT).

## Materials and methods

Study design and patients

This prospective interventional intent-to-treat study was conducted at the Department of Radiotherapy, American Hellenic Educational Progressive Association (AHEPA) University Hospital of Thessaloniki, and enrolled 23 newly diagnosed patients with histologically confirmed early glottic cancer (T1-T2N0M0) between June 2022 and August 2025. Eligible patients were >18 years old, Eastern Cooperative Oncology Group (ECOG) 0-2, without prior malignancy-oriented treatment, and with adequate organ function. Baseline CT or MRI was required to exclude nodal disease and assess tumor extent. Exclusion criteria were distant metastasis, concurrent malignancy, prior head and neck radiotherapy, major comorbidities, or contraindications to radiotherapy. Prospective data collection and analysis were approved by the Research Ethics Committee of the School of Medicine, Aristotle University of Thessaloniki, Faculty of Medicine (AHEPA University Hospital) under protocol number 341/2025 (meeting no. 5/14.03.2023), and all patients provided informed consent.

Radiotherapy technique

Patients were immobilized in the supine position with a thermoplastic mask for CT-based simulation. Target volumes were delineated according to published larynx contouring guidelines, with a 5-10 mm margin from gross tumor volume (GTV) to clinical target volume (CTV) and an additional isotropic 3-5 mm to define the planning target volume (PTV) [10]. Treatment plans were generated using volumetric modulated arc therapy (VMAT) with 6 MV photon beams delivered in a double full arc of 360°. Each arc started at 180° and rotated counterclockwise (CCW), with a gantry angle increment of 20°. The collimator and couch angles were set to 0°. Dose calculations were performed in the Monaco Treatment Planning System version 5.11.03 (Stockholm, Sweden: Elekta Ltd.), employing the Monte Carlo algorithm. A calculation grid size of 0.3 cm and a statistical uncertainty of 0.5% per calculation were used, with the dose deposition calculation mode set to medium to balance computational accuracy and efficiency.

Planning objectives required that V₉₅% of the PTV exceed 99%, ensuring adequate target coverage. The corresponding D₉₅% was set to at least 95% of the prescribed dose, and the mean PTV dose (Dmean) was equal to the prescription dose (55 Gy in 20 fractions; 2.75 Gy per fraction). Dose hotspots were allowed up to 105% of the prescribed dose.

All doses were converted to equivalent 2 Gy fractions (EQD₂) using the linear-quadratic (LQ) model without time correction, assuming an α/β ratio of 3 Gy for late-responding normal tissues. For the study regimen, this corresponds to an approximate EQD₂ of 63 Gy. Organ-at-risk (OAR) constraints were defined according to Quantitative Analyses of Normal Tissue Effects in the Clinic (QUANTEC)-based tolerance limits, expressed in EQD₂ terms as follows: spinal cord maximum ≤50 Gy, cervical esophagus V₄₅ <33%, and submandibular gland mean <35 Gy. These limits were selected to minimize the risk of late toxicity in head-and-neck radiotherapy [11].

Image guidance was performed using cone-beam computed tomography (CBCT) before each of the first three treatment fractions and subsequently every five fractions. On all other treatment days, kV portal imaging was used for verification. Positional deviations smaller than 1 mm in any translational direction were not corrected. Comprehensive quality assurance (QA) procedures were implemented for all treatment plans prior to delivery.

Evaluation criteria

Endpoints were toxicity and local control. Tumor response was assessed weekly during treatment, then at one month post-therapy, every three months for two years, and every six months thereafter, primarily based on laryngoscopic examination and imaging (CT or MRI) as indicated. Suspected recurrences were confirmed histologically, with imaging as indicated. Acute and late toxicities were graded according to the Radiation Therapy Oncology Group/European Organization for Research and Treatment of Cancer (RTOG/EORTC) criteria, with events occurring within three months of radiotherapy considered acute and those thereafter classified as late [12].

Statistical analysis

This phase I-II study was primarily designed to evaluate feasibility and safety; therefore, no formal power calculation was performed. The planned sample size of 20-25 patients was deemed adequate to detect clinically relevant acute or late toxicities with an incidence of ≥10-15% with reasonable confidence.

All analyses were conducted using SPSS version 23 (Armonk, NY: IBM Corp.). Survival and recurrence outcomes were calculated from the last day of radiotherapy. Endpoints included local control and overall survival (OS), estimated using the Kaplan-Meier method, with 95% confidence intervals (95% CIs) derived from the standard error of the survival estimate (estimate±1.96×SE).

Comparisons between T1 and T2 subgroups were predefined and performed using the log-rank test. Univariate Cox regression was applied to explore associations between local control and clinical or treatment-related variables, including smoking, alcohol use, histological differentiation, T stage, anterior commissure involvement, supra-, sub-, or trans-glottic extension, cord mobility, prior laser therapy, and overall treatment time (>32 vs. ≤32 days). Statistical significance was defined as p<0.05.

Local control was defined as the absence of persistent or recurrent disease at the primary site after completion of radiotherapy. Patients who achieved durable disease-free status following successful salvage for an initial partial response were considered locally controlled, whereas those who developed local recurrence after an initial complete response were classified as local failures. For Kaplan-Meier and Cox analyses, the first occurrence of persistent or recurrent disease was treated as an event, irrespective of subsequent salvage.

## Results

Patient characteristics

A total of 23 consecutive patients (22 males/one female) were enrolled between July 2022 and August 2025. Baseline patient characteristics are summarized in Table 1. All patients had biopsy-proven squamous cell carcinoma and had undergone a staging CT/MRI of the larynx. Three patients had undergone prior laser treatment (transoral laser surgery {TLS}) for early glottic carcinoma, and radiotherapy was delivered to these patients according to evidence of residual disease following laser treatment. Median duration of radiotherapy was 29 days (range: 28-39 days). Treatment duration was greater than 32 days in five patients; reason for delay was patients’ compliance for four patients, and not documented for one other patient. The median age at time of enrollment was 65 years (range: 49-87 years). There were 17 former smokers, and six who continued smoking after treatment. Stage distribution was 13 (56.5%) cT1a, two (8.7%) cT1b, and eight (34.8%) cT2.

**Table 1:** Patient and disease characteristics (n=23).

Characteristics	n (%)
Median age in years (range)	65 (49-87)
Gender	Male: 22 (95.7)
	Female: 1 (4.3)
Performance status	0-1: 15 (65.2)
	2: 8 (34.8)
Alcohol use (moderate or heavy)	Never: 8 (34.8)
	Former: 13 (56.5)
	Current: 2 (8.7)
Smoking	Never: 0 (0)
	Former: 17 (73.9)
	Current: 6 (26.1)
T-stage	cT1a: 13 (56.5)
	cT1b: 2 (8.7)
	cT2: 8 (34.8)
Differentiation	Well differentiated: 9 (39.1)
	Moderately differentiated: 12 (52.2)
	Poorly differentiated: 2 (8.7)
Extension	Supraglottic: 5 (21.7)
	Subglottic: 3 (13)
	Transglottic: 0 (0)
Anterior commissure involvement	Involved: 11 (47.8)
	Noninvolved: 12 (52.2)
Cord mobility	Mobile: 16 (69.6)
	Impaired: 7 (30.4)
Prior laser	Yes: 3 (13)
	No: 20 (87)

Treatment outcomes

The median follow-up duration was 16 months (range: 1-36 months). A complete clinical response to radiotherapy was achieved in 22 of 23 patients (95.7%), while one patient with stage T1bN0M0 glottic carcinoma exhibited residual disease, underwent transoral laser surgery (TLS), and remained disease-free at the last follow-up.

Disease recurrence was observed in two patients who had initially achieved a complete response. The first case (T2N0M0 with subglottic extension and anterior commissure involvement) recurred six months after radiotherapy and was managed with salvage laryngectomy. The second case (T1aN0M0) recurred seven months after treatment and was successfully salvaged with TLS. Both patients remained disease-free at the last follow-up and had continued smoking during radiotherapy.

**Figure 1:**
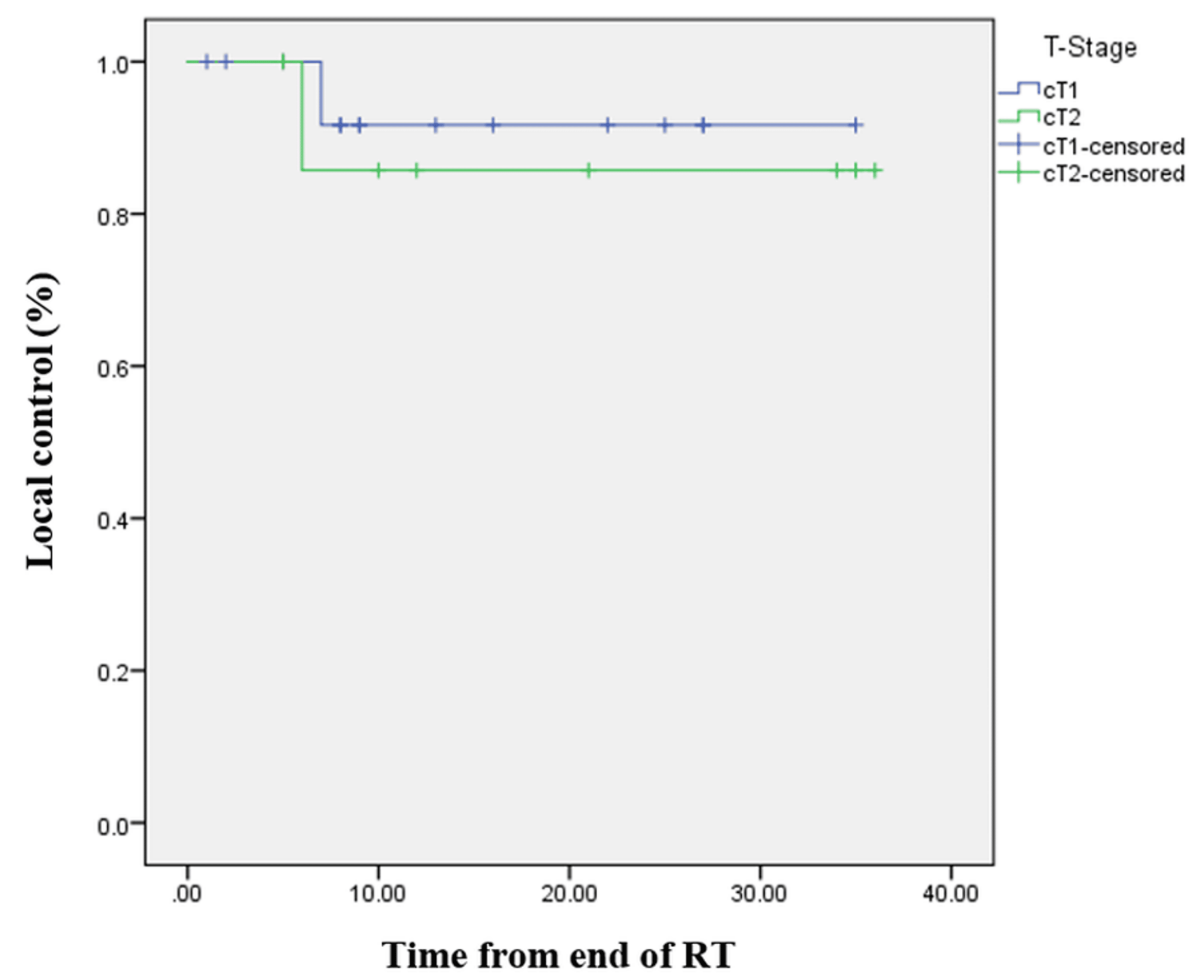
Kaplan-Meier curve showing local control according to stage. RT: radiotherapy

According to the predefined criteria, the observed local control was 91.3% overall (93.3% for T1 and 87.5% for T2 disease). By Kaplan-Meier analysis, the estimated one-year local control was 91.7% for T1 (95% CI: 76.0-100.0%) and 85.7% for T2 (95% CI: 59.8-100.0%), with no significant difference between subgroups (log-rank p=0.65). Continued smoking during radiotherapy was associated with inferior local control on Kaplan-Meier analysis (log-rank p=0.012). However, the corresponding univariate Cox regression yielded an unstable hazard ratio (HR: 492.3; 95% CI: 0-1.8×10¹⁰; p=0.486), reflecting the limited number of recurrence events and small cohort size. No other covariate demonstrated a statistically significant association with local control (all p>0.05). Seventeen of 23 patients (73.9%) completed radiotherapy within 32 days; one of the two recurrences occurred in a patient whose overall treatment time exceeded 32 days, but treatment duration was not significantly associated with local control (p=0.52). Figure 1 illustrates local control in relation to tumor stage. No deaths occurred during follow-up.

Toxicity and safety

Details of acute and late toxicities are summarized in Table 2. According to RTOG criteria, most acute toxicities involving the larynx, mucous membranes, skin, and esophagus were grade 1-2. Three patients developed grade 3 acute esophageal toxicity (dysphagia) requiring temporary enteral nutrition, and seven patients experienced grade 3 acute laryngeal toxicity (hoarseness), which was managed with low-dose dexamethasone. No grade 4 acute events were observed. Late toxicities were predominantly grade 1 or less. One patient developed grade 2 skin toxicity, one patient grade 2 esophageal toxicity (dysphagia), and five patients grade 2 laryngeal toxicity (persistent hoarseness). No cases of second primary malignancies were observed during follow-up. For two patients, only acute toxicity was recorded, as their follow-up was shorter than three months.

**Table 2:** Acute and late toxicities.

Toxicity site	Acute toxicity, n (%)			Late toxicity, n (%)		
	Grade 1	Grade 2	Grade 3	Grade 1	Grade 2	Grade 3
Skin	18 (78.3)	5 (21.7)	0 (0)	6 (28.6)	1 (4.8)	0 (0)
Esophagus	7 (30.5)	13 (56.5)	3 (13)	7 (33.3)	1 (4.8)	0 (0)
Mucous membrane	8 (34.8)	5 (21.7)	0 (0)	3 (14.3)	0 (0)	0 (0)
Larynx	3 (13)	13 (56.5)	7 (30.5)	16 (76.2)	5 (23.8)	0 (0)

Comparison with historical controls

In our previous institutional clinical audit of 57 patients with early-stage glottic carcinoma treated primarily with conventional or mildly hypofractionated three-dimensional conformal radiotherapy, the five-year local control rate was 86% overall, with higher control for T1 (90.9%) compared to T2 disease (79.2%) [13]. Acute and late toxicities were generally mild, although a small proportion of patients experienced grade 3 mucositis or hoarseness, and severe late toxicity was rare.

Compared with the current prospective series, the more hypofractionated VMAT-based schedule achieved at least comparable early local control (91.3% overall; 93.3% for T1 and 87.5% for T2 disease) and showed a similarly favorable toxicity profile. These findings suggest that modern image-guided VMAT delivery may provide equivalent or improved efficacy with excellent tolerance in early glottic cancer, while maintaining treatment feasibility and safety.

## Discussion

Dose fractionation and overall treatment time are well-established determinants of outcome in definitive radiotherapy for early-stage glottic carcinoma. Clinical data from head and neck cancer suggest that local tumor control decreases substantially with prolongation of treatment, by an estimated 3-25% (median 15%) after one additional week, and 5-42% (median 26%) after two additional weeks of treatment [4]. This effect is attributed to accelerated repopulation of clonogenic tumor cells, which typically commences after approximately three to four weeks of conventionally fractionated treatment and represents a critical factor influencing local control. To compensate for this, an additional dose of 0.4-1.2 Gy per day of treatment extension is considered necessary [5,6]. Hypofractionation is particularly attractive in this context, as it reduces overall treatment time and minimizes the likelihood of repopulation during therapy, while also potentially overcoming the relative radioresistance of epithelial tumors, consistent with their potentially low α/β ratio in the linear-quadratic (LQ) model [7,14].

Clinical evidence supports hypofractionated schedules in early-stage glottic carcinoma, with randomized trials showing superior or at least comparable outcomes for T1 disease using 2.25 Gy per fraction [15,16]. Large retrospective series also suggest potential benefit, though results for T2 tumors are less consistent [17,18]. More accelerated regimens with larger fraction sizes (>2.5 Gy) have yielded local control rates of 88-93% in T1 disease, broadly comparable or superior to conventional fractionation [19-22]. Together, these studies suggest that hypofractionation is safe and effective for T1 glottic carcinoma, while its role in T2 disease, though encouraging, requires further validation.

Our prospective evaluation of a hypofractionated VMAT regimen delivering 55 Gy in 20 fractions (2.75 Gy per fraction) demonstrated encouraging early outcomes. With a median follow-up of 16 months, the Kaplan-Meier one-year local control was 91.3% overall, 91.7% for T1, and 85.7% for T2 disease, consistent with previously reported results for early glottic carcinoma. Acute and late toxicities were mild, with few grade 3 events and no grade 4 toxicity, confirming the feasibility and safety of this regimen. These results support the effectiveness of moderate hypofractionation using highly conformal VMAT delivery. When compared with our prior institutional experience using conventional or mildly hypofractionated three-dimensional conformal radiotherapy, the present VMAT-based approach demonstrated at least comparable early disease control and a similarly favorable toxicity profile. This suggests that advances in treatment planning and delivery may contribute to maintaining efficacy while improving treatment efficiency and overall patient convenience, with the shorter regimen potentially enhancing treatment adherence and quality of life. Overall, these findings underscore the feasibility of integrating hypofractionated VMAT regimens into routine practice for early-stage glottic carcinoma.

This study has several limitations. First, the median follow-up of 16 months is relatively short, limiting the ability to assess long-term disease control and late toxicity. Second, the sample size is modest, and no formal power calculation was performed, given the feasibility design, which restricts statistical power, particularly for subgroup analyses between T1 and T2 disease. Accordingly, the findings in the T2 subgroup should be considered exploratory and may be underpowered to demonstrate equivalence with conventional fractionation. Third, while all patients in this study were treated uniformly with VMAT and a single hypofractionated schedule, comparisons with institutional historical cohorts treated with three-dimensional conformal radiotherapy (3D-CRT) should be interpreted with caution, as differences in patient selection, staging, and treatment era may introduce bias. The single-institution design also limits the generalizability of the results. Furthermore, the apparent association between continued smoking during radiotherapy and inferior local control, although significant on log-rank testing, should be interpreted cautiously, given the wide confidence interval and the small number of recurrence events. Finally, the absence of patient-reported outcomes such as voice and swallowing function limits the evaluation of functional endpoints that are particularly relevant in early glottic cancer. Longer follow-up and prospective validation in larger multicenter cohorts will be necessary to confirm the durability and reproducibility of these findings.

## Conclusions

This prospective study demonstrates that hypofractionated radiotherapy using 55 Gy in 20 fractions delivered with VMAT and daily image guidance is a safe and effective regimen for patients with early-stage glottic carcinoma. Our results confirm very good local control for T1 tumors and acceptable outcomes for T2 disease, with manageable acute and late toxicities. Compared with conventional and mildly hypofractionated schedules reported in the literature and in our prior institutional audit, this more hypofractionated regimen achieves comparable efficacy with the advantage of reducing overall treatment time. While the relatively short follow-up limits definitive conclusions on long-term control and late effects, our findings contribute valuable prospective data supporting the role of hypofractionation in the management of early glottic cancer.

## References

[1] Sung H, Ferlay J, Siegel RL, Laversanne M, Soerjomataram I, Jemal A, Bray F (2021). Global Cancer Statistics 2020: GLOBOCAN estimates of incidence and mortality worldwide for 36 cancers in 185 countries. CA Cancer J Clin.

[2] Huang J, Chan SC, Ko S (2024). Updated disease distributions, risk factors, and trends of laryngeal cancer: a global analysis of cancer registries. Int J Surg.

[3] Silver CE, Beitler JJ, Shaha AR, Rinaldo A, Ferlito A (2009). Current trends in initial management of laryngeal cancer: the declining use of open surgery. Eur Arch Otorhinolaryngol.

[4] Overgaard J, Hansen HS, Specht L (2003). Five compared with six fractions per week of conventional radiotherapy of squamous-cell carcinoma of head and neck: DAHANCA 6&7 randomised controlled trial. Lancet.

[5] Skladowski K, Tarnawski R, Maciejewski B (1999). Clinical radiobiology of glottic T1 squamous cell carcinoma. Int J Radiat Oncol Biol Phys.

[6] Le QT, Fu KK, Kroll S (1997). Influence of fraction size, total dose, and overall time on local control of T1-T2 glottic carcinoma. Int J Radiat Oncol Biol Phys.

[7] Yamazaki H, Suzuki G, Nakamura S, Yoshida K, Konishi K, Teshima T, Ogawa K (2017). Radiotherapy for laryngeal cancer - technical aspects and alternate fractionation. J Radiat Res.

[8] Mayo ZS, Ilori EO, Matia B (2022). Limited toxicity of hypofractionated intensity modulated radiation therapy for head and neck cancer. Anticancer Res.

[9] (2024). Radiotherapy Dose Fractionation. Fourth Edition. Radiologists.

[10] Choi M, Refaat T, Lester MS, Bacchus I, Rademaker AW, Mittal BB (2014). Development of a standardized method for contouring the larynx and its substructures. Radiat Oncol.

[11] Marks LB, Yorke ED, Jackson A (2010). Use of normal tissue complication probability models in the clinic. Int J Radiat Oncol Biol Phys.

[12] Cox JD, Stetz J, Pajak TF (1995). Toxicity criteria of the Radiation Therapy Oncology Group (RTOG) and the European Organization for Research and Treatment of Cancer (EORTC). Int J Radiat Oncol Biol Phys.

[13] Moschos G, Theodoridou A, Stylianidou S, Capizzello A, Plataniotis G (2023). Radical radiotherapy for early-stage carcinoma of the glottis. Should we switch to hypofractionation?. Biomed J Sci Tech Res.

[14] Qi XS, Yang Q, Lee SP, Li XA, Wang D (2012). An estimation of radiobiological parameters for head-and-neck cancer cells and the clinical implications. Cancers (Basel).

[15] Yamazaki H, Nishiyama K, Tanaka E, Koizumi M, Chatani M (2006). Radiotherapy for early glottic carcinoma (T1N0M0): results of prospective randomized study of radiation fraction size and overall treatment time. Int J Radiat Oncol Biol Phys.

[16] Moon SH, Cho KH, Chung EJ (2014). A prospective randomized trial comparing hypofractionation with conventional fractionation radiotherapy for T1-2 glottic squamous cell carcinomas: results of a Korean Radiation Oncology Group (KROG-0201) study. Radiother Oncol.

[17] Kim TG, Ahn YC, Nam HR, Chung MK, Jeong HS, Son YI, Baek CH (2012). Definitive radiation therapy for early glottic cancer: experience of two fractionation schedules. Clin Exp Otorhinolaryngol.

[18] Mendenhall WM, Amdur RJ, Morris CG, Hinerman RW (2001). T1-T2N0 squamous cell carcinoma of the glottic larynx treated with radiation therapy. J Clin Oncol.

[19] Gowda RV, Henk JM, Mais KL, Sykes AJ, Swindell R, Slevin NJ (2003). Three weeks radiotherapy for T1 glottic cancer: the Christie and Royal Marsden Hospital Experience. Radiother Oncol.

[20] Cheah NL, Lupton S, Marshall A, Hartley A, Glaholm J (2009). Outcome of T1N0M0 squamous cell carcinoma of the larynx treated with short-course radiotherapy to a total dose of 50 Gy in 16 fractions: the Birmingham experience. Clin Oncol (R Coll Radiol).

[21] Laskar SG, Baijal G, Murthy V, Chilukuri S, Budrukkar A, Gupta T, Agarwal JP (2012). Hypofractionated radiotherapy for T1N0M0 glottic cancer: retrospective analysis of two different cohorts of dose-fractionation schedules from a single institution. Clin Oncol (R Coll Radiol).

[22] Short S, Krawitz H, Macann A (2006). T1N0/T2N0 glottic carcinoma: a comparison of two fractionation schedules. Australas Radiol.

